# Antimicrobial Drug Use and Antibiotic-Resistant Bacteria

**DOI:** 10.3201/eid1401.071028

**Published:** 2008-01

**Authors:** Fernando Bellissimo-Rodrigues

**Affiliations:** * Hospital das Clínicas da Faculdade de Medicina de Ribeirão Preto, São Paulo, Brazil

**Keywords:** antimicrobial use, antibiotic-resistant bacteria, infection control, hand hygiene, letter

## Abstract

Antimicrobial Drug Use and Antibiotic-Resistant Bacteria (response)

## To the Editor

The article by Harris et al., published in the August 2007 issue of Emerging Infectious Diseases, examined the risk factors for selecting extended-spectrum β-lactamase–producing *Enterobacteriaceae* in intensive-care patients and found that exposure to piperacillin/tazobactam and vancomycin were independent risk factors (*1*). Although antimicrobial drug use has been historically linked to antibiotic resistance in bacteria, we should not miss the perspective that such a risk factor mostly favors the cross-transmission of preexisting antibiotic-resistant bacteria, taking into account the disruption of the endogenous microflora, rather than the selection of “de novo” resistant mutants (*2*). This supposition is supported by many articles that have found genetic similarity between antibiotic-resistant microorganisms that occur in hospitalized patients, as well as by the fact that most of these pathogens exhibit cross-resistance with different classes of drugs, which should be extremely rare on a mutation basis.

This hypothesis is also supported by the evidence that healthcare workers frequently do not obey simple infection control precautions such as practicing hand hygiene between contact with different patients (*3*–*6*). That is likely why Larson et al., in a multicenter study in the United States, recently found no relationship between antimicrobial drug control policies and level of antibiotic resistance in bacteria, but did find an association between lower levels of antibiotic resistance in *Staphylococcus aureus* and enterococci and high compliance with hand hygiene (*7*).

Therefore, perhaps we should start looking for risk factors for being colonized or infected by any antimicrobial drug–resistant bacterium, including in our analysis some infection control measures adopted commonly during outbreak investigations, such as exposure to doctor A or nurse B, proximity to a known colonized patient, understaffing during the period of the study, and so forth. If we do so, we will likely find that antimicrobial drug use is not a completely independent risk factor for the mentioned outcome, but a risk factor closely related to the availability of the antibiotic-resistant microorganism in the local environment or on our own hands.
