# Supplementary material for: Antimicrobial Drug Use and Antibiotic-Resistant Bacteria
Source: Emerg Infect Dis. 2008 Jan;14(1):187–8. doi: 10.3201/eid1401.071028 (PMC2600174; doi:10.3201/eid1401.071028)
Supplement: Related texts

# Reply: In Response

**Authors:** Anthony D. Harris, Kerri Thom, Jessina McGregor

**Affiliations:** * University of Maryland, Baltimore, Maryland, USA; † Oregon State University, Portland, Oregon, USA

We appreciate the comments by Dr. Bellissimo-Rodrigues regarding our article analyzing risk factors for surveillance-culture positivity with extended-spectrum β-lactamase (ESBL)–producing bacteria (*1*). We agree with the author that patient-to-patient transmission is a potentially important causal factor in the emergence of resistance for ESBL-producing bacteria as well as for other antimicrobial drug–resistant bacteria, such as vancomycin-resistant enterococci, methicillin-resistant *Staphylococcus aureus*, and drug-resistant *Pseudomonas* and *Acinetobacter* spp. For each of these resistant organisms, a complicated interplay likely exists between the causal importance of antibiotic selective pressure and patient-to-patient transmission by healthcare workers (*2*–*4*). The relative importance of these 2 causal mechanisms needs to be determined for each individual antimicrobial-resistant bacterium. The relative causal importance may be different for different outcomes: colonization on admission, colonization acquisition, and progression from colonization to infection. Understanding the relative importance for each of these outcomes is needed before determining whether infection control interventions or antimicrobial drug stewardship policies will be effective. For example, we have published work that demonstrates that patient-to-patient transmission is important for colonization acquisition of ESBL-producing *Klebsiella* spp. and *Escherichia coli* (*5*).

We believe that a couple of points require clarification. In our article, we looked at risk factors for colonization with ESBL-producing bacteria on admission to an intensive care unit. We did not examine risk factors for selecting ESBL-producing bacteria as the letter implies. For the antimicrobial drugs identified as potential risk factors, we clearly stated in the discussion that the risk factors identified may be causally related to the outcome of ESBL-colonization or may only be statistically associated. We argue that even risk factors identified are not causal; they may be important because they can help determine which patients may need empiric antimicrobial drug therapy targeted to the ESBL-producing bacteria. Future research work is still needed to assess the relative importance of patient-to-patient transmission versus antimicrobial selective pressure.

## References

[1] Bellissimo-Rodriques F. Antimicrobial drug use and antibiotic-resistant bacteria. Emerg Infect Dis. 2008;14:187. 10.3201/eid1401.07102818258109PMC2600174

[2] Harris AD, McGregor JC, Furuno JP. What infection control interventions should be undertaken to control multidrug-resistant gram-negative bacteria? Clin Infect Dis. 2006;43(Suppl 2):S57–61. 10.1086/50447916894516

[3] Lipsitch M, Bergstrom CT, Levin BR. The epidemiology of antibiotic resistance in hospitals: paradoxes and prescriptions. Proc Natl Acad Sci U S A. 2000;97:1938–43. 10.1073/pnas.97.4.193810677558PMC26540

[4] Lipworth AD, Hyle EP, Fishman NO, Nachamkin I, Bilker WB, Marr AM, Limiting the emergence of extended-spectrum Beta-lactamase-producing enterobacteriaceae: influence of patient population characteristics on the response to antimicrobial formulary interventions. Infect Control Hosp Epidemiol. 2006;27:279–86. 10.1086/50301616532416

[5] Harris AD, Kotetishvili M, Shurland S, Johnson JA, Morris JG, Nemoy LL, How important is patient-to-patient transmission in extended-spectrum beta-lactamase *Escherichia coli* acquisition. Am J Infect Control. 2007;35:97–101. 10.1016/j.ajic.2006.09.01117327188
